# Salivary Oxytocin and Antioxidative Response to Robotic Touch in Adults with Autism Spectrum Disorder

**DOI:** 10.3390/ijms241512322

**Published:** 2023-08-01

**Authors:** Galina V. Portnova, Elena V. Proskurnina, Ivan V. Skorokhodov, Svetlana V. Sokolova, Alexey N. Semirechenko, Anton A. Varlamov

**Affiliations:** 1 Laboratory of Human Higher Nervous Activity, Institute of Higher Nervous Activity and Neurophysiology of the Russian Academy of Sciences, 5A Butlerova Str., 117485 Moscow, Russia; 2 Tactile Communication Research Laboratory, Pushkin State Russian Language Institute, 6 Volgina Str., 117485 Moscow, Russia; 3 Laboratory of Molecular Biology, Research Centre for Medical Genetics, 1 Moskvorechye Str., 115522 Moscow, Russia; proskurnina@med-gen.ru; 4 Autonomous Non-Profit Organization “Our Sunny World”, 98 Nizhegorodskaya Str., 109052 Moscow, Russia; 5 Medical Scientific and Educational Center, Lomonosov Moscow State University, Lomonosovsky Prosp. 27-10, 119991 Moscow, Russia

**Keywords:** saliva, oxytocin, antioxidants, tactile stroke, electroencephalography, autism spectrum disorder

## Abstract

Individuals with ASD are known to have a tendency to have tactile sensory processing issues that could be associated with their impairment as regards social communication. The alterations in tactile processing in autistic subjects are usually accompanied by hypersensitivity and other unpleasant emotions induced by tactile contact. In our study, we investigated the impact of the velocity and the force of a tactile stroke received impersonally by a custom-built robotic device. A total of 21 adults with ASD and 22 adults from a control group participated in our study. The participants’ responses were assessed according to subjective scales, EEG changes, and the dynamics of saliva antioxidants and oxytocin. It was found that the oxytocin level was significantly lower in subjects with ASD but increased after tactile stimulation. However, contrary to expectations, the increase in the oxytocin level in the target group negatively correlated with the subjective pleasantness of tactile stimulation and was probably associated with a stress-induced effect. The basic levels of antioxidants did not differ between the TD and ASD groups; however, these had significantly increased in individuals with ASD by the end of the study. The EEG findings, which revealed enhanced antioxidant levels, contributed to the relief of the cognitive control during the study.

## 1. Introduction

Autism spectrum disorder (ASD) is a generalized neurodevelopmental disorder that is characterized by variable communication deficits, repetitive behaviors, and restrictive interests [1]. The pathogenesis of ASD is very complex, but there is no doubt it includes an abnormality of the brain structure and function, as has been verified using modern instrumental methods (electroencephalography, functional magnetic resonance imaging, diffusion tensor imaging, and near-infrared optical imaging) [2]. Abnormalities in biochemical and neuromediator systems are also common in ASD [3].

The metabolism of oxytocin and serotonin is also altered in ASD; while oxytocin deficiency or receptor dysfunction are associated with social communication disorders, serotonin disfunction is associated with repetitive behaviors [4]. Oxytocin is a hormone that plays the role of a “social key” [5]. Autistic children have lower levels of oxytocin than typically developing (TD) children [6]. However, this phenomenon has not been verified for adolescents and adults, although males with ASD tend to have decreased oxytocin levels [7]. However, instrumental studies have proven the direct involvement of oxytocin in the regulation of social interaction in autism [8,9]. Oxytocin treatment has been successfully used in animals and patients with ASD [10,11]. Interestingly, the administration of exogenous oxytocin somehow stimulates a steady increase in endogenous oxytocin [12]. However, there is evidence that oxytocin administration in ASD does not improve one’s emotional status and social skills [13], which seem to be related to the complex pathogenesis of autism.

Asperger’s syndrome is currently included in the autism spectrum, being considered a variant of high-functioning ASD without intellectual disorders [14]. Di Napoli et al. reported the genetic variation in the oxytocin receptor gene associated with Asperger’s syndrome [15]. In Asperger’s syndrome, the administration of oxytocin resulted in a significant reduction in repetitive actions [16]. Intranasal administration of oxytocin improved the recognition of facial emotions in people with Asperger’s syndrome [17]. All of the above proves the need for and importance of a systematic study on the role of oxytocin in the pathogenesis of ASD and Asperger’s syndrome.

The balance of reactive oxygen species (ROS) plays a key role in the pathogenesis of almost all diseases, including psychiatric disorders. Oxidative stress reflects an imbalance between the production of reactive oxygen species and the neutralizing ability of the antioxidant network. Oxidative stress accompanies psychoemotional stress, and the administration of antioxidants in emotional pain stress prevents vegetative disorders, cardiac hypertrophy, and oxidative damage to the brain in rats [18]. In psychoemotional stress, the antioxidant status of saliva is more relevant than changes in blood plasma are [19]. Saliva is a promising object in laboratory diagnostics, especially for patients with psychiatric disorders, since it can be collected painlessly without additional stress. In our previous studies, we examined the antioxidant status of saliva and oxytocin in response to pleasant touch in TD volunteers [20].

Long-term oxidative stress is one of the main factors contributing to cognitive deficit concurrent with aging. Antioxidant intake favorably affects the memory and cognitive functions of the elderly and older animals [21,22]. However, data on the effectiveness of the antioxidant diet can be contradictory [23,24], which indicates the need for a more thorough study of the biochemical mechanisms of cognitive impairment. However, in general, authors discuss the benefits of antioxidant therapy for the correction of cognitive functions [25,26,27]. Oxidative stress plays an undeniable role in the pathogenesis of ASD [28,29,30,31]. The beneficial effects of antioxidants on ASD symptoms have been noted in many studies [32], especially for antioxidants with anti-inflammatory properties [33]. Diets aimed at increasing glutathione are particularly effective [34], which is consistent with the results of studies demonstrating glutathione deficiency in ASD [35,36]. Oxidative stress in Asperger’s syndrome has not been studied so intensively. For adolescents, a decrease in the total antioxidant status, the level of copper and ceruloplasmin, and an increase in the level of homocysteine have been shown [37].

In addition to the standardized tests for ASD diagnostics, including scales such as ADOS-2 [38], researchers continue to search for biomarkers for objective diagnostics. The electrophysiological, hormonal, and biochemical specific parameters could provide a less subject-dependent approach to ASD diagnostics [39]. At the same time, all clinical diagnostic procedures, including EEG and blood collection in children with autism, verify the biochemical and electroencephalographic changes associated with emotional stress with corresponding EEG, biochemical, hormonal, and EEG abnormalities [20,40,41,42]. Other studies show that any atypical situation (e.g., a long road trip) may cause anxiety and related stress [43], which can be seen both at behavioral [44] and physiological levels, reflected in skin conductance and heart rate variability. In clinical settings, a novel sensory environment plays a significant role in developing a stress reaction, as people with ASD encounter new sensations, including a tight EEG cap and slimy conductive gel in their hair.

Until recently, perception abnormalities in autism were overlooked, as researchers were looking into behavioral and cognitive issues. However, in the late XX century, another trend emerged, which tries to comprehend autism as a condition caused by abnormal perception [45,46]. As tactile perception is linked to rewarding social communication, there is a hypothesis of tactile aversion contribution to ASD phenotype development [47].

Up to the current day, tactile perception abnormalities in ASD as well as their biochemical and electrophysiological correlates remain understudied. One of the reasons for this is the above-described difficulties of contact with subjects with ASD and their limited ability to describe their subjective sensations [48]. In our study, this issue was addressed by inviting highly functional adult subjects with ASD [49] and the application of the rotary tactile stimulator (RTS) to deliver tactile stimuli with a strict control of their velocity, intensity, or pressing force of stroke [50,51,52]. The use of a robotic system to deliver tactile stimuli significantly reduces contact with another person, which is one of the most stressful factors for subjects with ASD [53]. In this situation, the tactile stimulus is separated from its social component, and we were able to investigate its direct cognitive and emotional reaction in subjects with ASD. Another innovation of the research was to compare antioxidant and hormonal (oxytocin) changes with EEG changes in response to tactile stimulation in subjects with ASD, which has not been performed previously in a single study. The study of antioxidant capacity and oxytocin, which are fundamentally different systems but both related to emotional stress; a comparison of them both with EEG dynamics at rest and during the presentation of tactile stimuli; and a comparison of them with subjective sensations allow a better understanding of the nature of sensory disorders in subjects with ASD.

## 2. Results

### 2.1. Subjective Assessment of Stimuli

**Figure 1:**
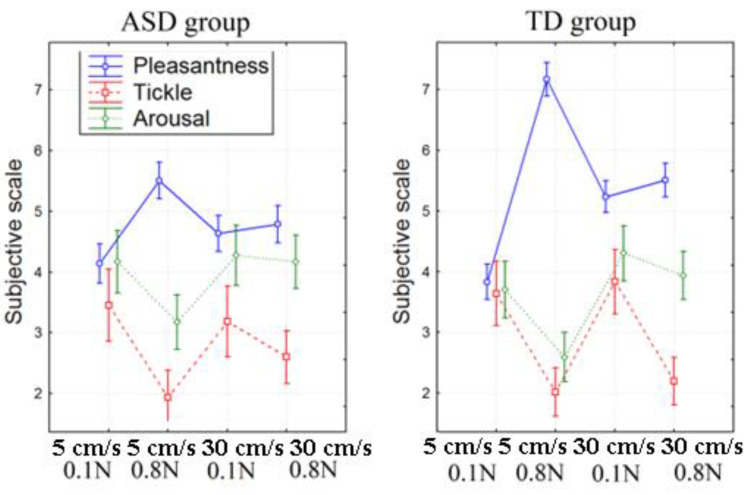
Subjective rating of different types of strokes by groups.

The subjects were asked to rate each type of stimulation in terms of their pleasantness, ticklishness, and arousal. TD subjects rated each type of touch as more pleasant in comparison with their ASD peers (F(1, 42) = 8.8785, *p* = 0.00501, partial eta-squared = 0.19). Both groups rated slow strokes with moderate force (0.8 N) as the most pleasant ones (F(3, 123) = 23.434, *p* < 0.000001, partial eta-squared = 0.38). TD subjects rated slow light (>0.1 N) strokes as the least pleasant (F(3, 114) = 4.0660, *p* = 0.00873, partial eta-squared = 0.1), while ASD participants did not distinguish it from other types of stroking. Both groups rated light strokes as the most ticklish ones (F(3, 114) = 17.878, *p* < 0.000001, partial eta-squared = 0.32). Differences between groups formed according to their antioxidant responses were not significant. Detailed results are presented in Table 1 and Figure 1.

### 2.2. Salivary Oxytocin and Antioxidant Levels

Antioxidant capacity rose after tactile stimulation (time effect F(1, 36) = 18.489, *p* = 0.00012, Partial eta-squared = 0.34). A post hoc Bonferroni test showed that this change was significant only in the ASD group (group × time effect F(1, 36) = 6.7253, *p* = 0.01366, partial eta-squared = 0.16, Bonferroni test MSE = 3163E2, df = 48,485: time effect for Asperger’s *p* = 0.000193, for control group *p* = 1). ASD participants also had a significantly higher antioxidant index (AI) (*t*-value = 2.67, *p* = 0.01).

**Figure 2:**
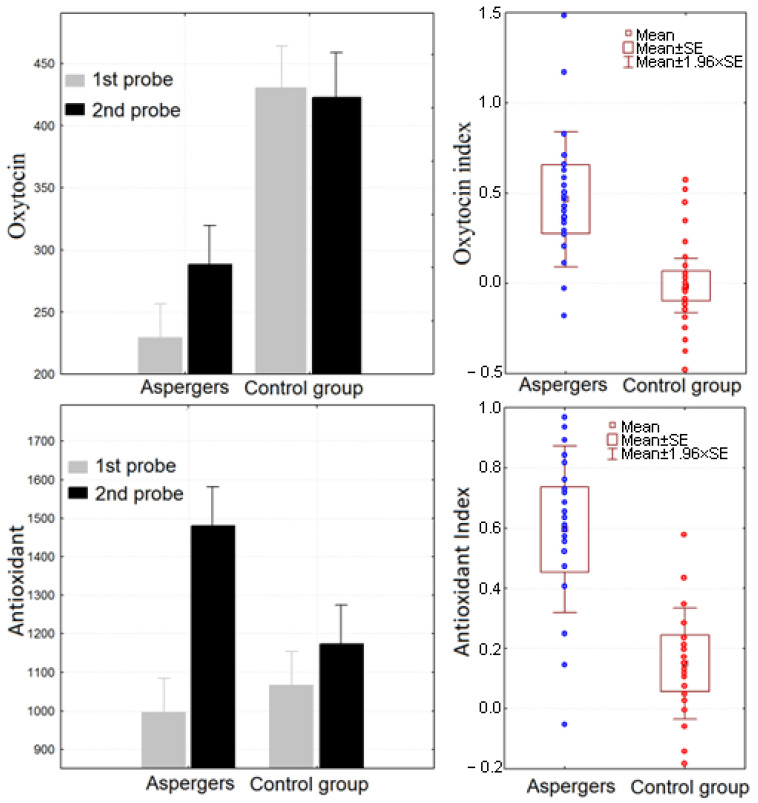
The level of the oxytocin and antioxidant capacity in TD and ASD group and individual values of antioxidant or oxytocin indices in two groups of subjects. Blue circles—participants with ASD; red circles—TD participants.

Oxytocin levels were significantly higher in TD participants (group effect F(1, 36) = 12.279, *p* = 0.00124, partial eta-squared = 0.25), but only ASD participants showed an oxytocin response to tactile stimulation (F(1, 36) = 5.3720, *p* = 0.02626, partial eta-squared = 0.13); the oxytocin index (OI) was higher in the ASD group as well (*t*-value = 2.65, *p* = 0.01). See Table 2 and Figure 2 for detailed results.

### 2.3. Electroencephalographic Findings

#### 2.3.1. Peak Alpha Frequency (PAF)

**Figure 3:**
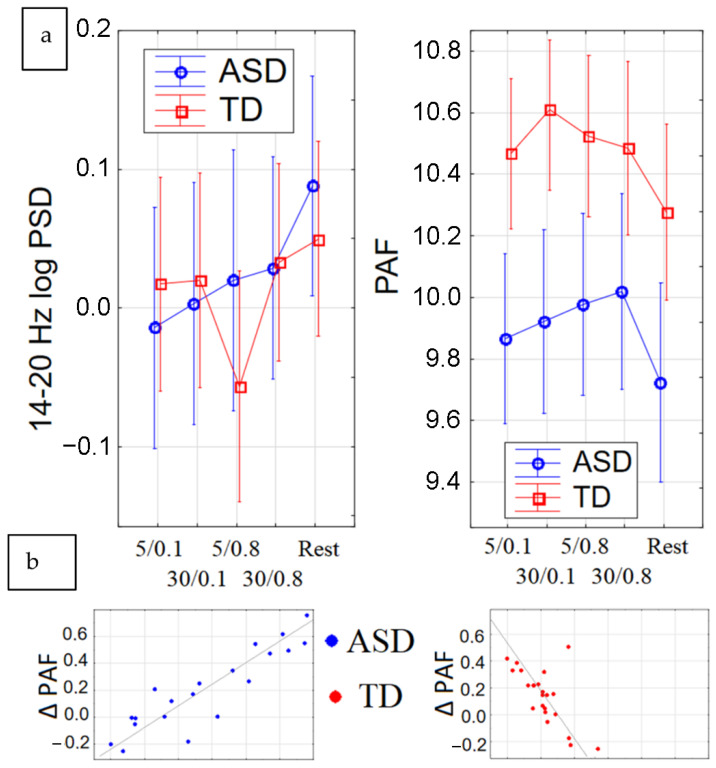
(**a**) dynamics in beta power towards different strokes, (**b**) PAF changes by groups.

The PAF was significantly higher in all the analyzed electrodes in the TD group (group effect F(1, 42) = 7.0147, *p* = 0.01161, partial eta-squared = 0.15). As shown in Figure 3b, both groups showed a significant PAF increase towards stroking in comparison with the resting state for all types of stimulation, as shown with the post hoc Bonferroni test (post hoc Bonferroni test, *p* < 0.038). No differences in PAF were found for the Yes/No groups.

#### 2.3.2. Beta Power

Both groups showed a significant power increase in the beta range (14–20 Hz). According to the post hoc Bonferroni test, the differences related to the rest state were significant for all types of stimuli in the ASD group (post hoc Bonferroni test, *p* < 0.0078), while the TD group showed a significant change (*p* = 0.0015) in beta power only towards slow and mild stimulation (5 cm/s, 0.8 N), which has optimal force and velocity for C-tactile afferent activation (Figure 3a).

#### 2.3.3. Event-Related Potentials (ERPs)

**Figure 4:**
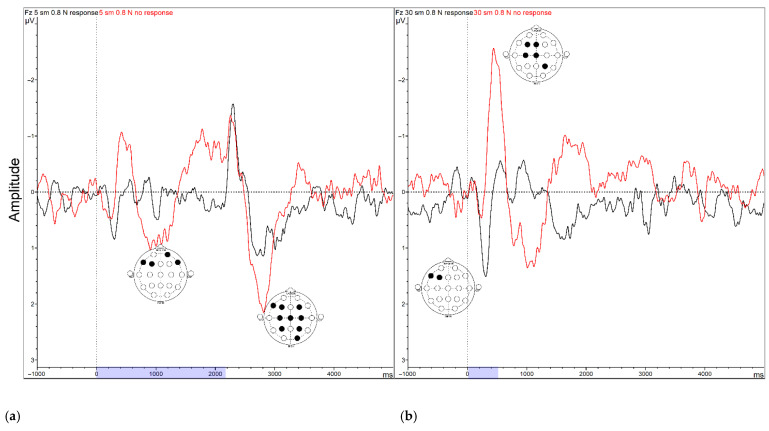
(**a**) ERP waveforms for slow intense strokes, (**b**) ERP waveforms for fast intense strokes. Red curve corresponds to the No group; black curve corresponds to *Yes* group. Blue section on the timescale shows the time of stimulation (from first touch to full brush offset). The electrodes where the differences for specific ERP components were found are shown on the minimaps with black filling.

The amplitudes of ERPs for stimulation onset (for all stimuli) and offset (for slow strokes only) were significantly higher in the group with a distinct antioxidant response (group effect F(1, 41) = 9.4050, *p* = 0.00382, partial eta-squared = 0.26). This was found both in ASD and TD participants. Pairwise post hoc *t*-tests allowed us to locate the electrodes where the differences in amplitude were statistically significant. ERP curves and EEG maps are shown in Figure 4.

#### 2.3.4. Resting-State EEG Dynamics

We compared the resting-state EEG recorded before stimulation and in interstimulus intervals (about 4 s) in all the three blocks.

We did not find significant group differences in EEG dynamics between the TD and ASD groups; however, we found significant changes in the EEG parameters measured during resting-state intervals in the beginning and at the end of the study as well as between blocks and between subjects according to their antioxidant response to the stimulation (Yes/No groups). In particular, the participants of the Yes group demonstrated a significant decrease in the theta rhythm in the second and third block compared to the resting state in the very beginning of the study and the first block (time × group effect F(3, 123) = 8.9936, *p* = 0.00002). The effect was most prominent in the central and parietal regions (time × topography × group effect F(6, 246) = 4.3256, *p* = 0.00036). The results of the post hoc analysis are presented in Table 3. The fractal dimension (FD) decreased from the beginning to the end of the study in the Yes group (time × group effect F(3, 123) = 14.7763, *p* < 0.00001).

### 2.4. Correlation between Subjective Ratings and Biochemical Response

**Figure 5:**
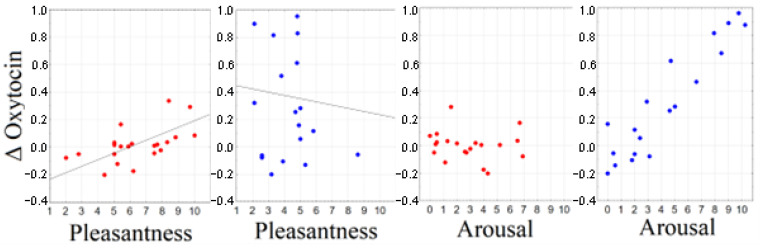
Scatterplots of association between oxytocin index and subjective assessments. Blue circles—participants with ASD; red circles—TD participants.

The correlation between the increase in salivary oxytocin levels and subjective ratings of stimuli differed between the ASD and TD groups. Thus, an increase in oxytocin in TD subjects correlated with higher scores on the “pleasantness” scale (*r* > 0.66, *p* < 0.005), whereas subjects with ASD had higher scores on the “arousal” scale (*r* > 0.61, *p* < 0.007). This phenomenon was most pronounced for the stimulus with the C-tactile optimal force and velocity (see Figure 5).

### 2.5. Correlation between Electrophysiological and Biochemical Response

The correlation between the increase in oxytocin levels and EEG changes corresponding to tactile stimuli in the ASD and TD groups had an opposite tendency. Thus, when a slow and intense stimulus was presented, the control group showed a significant inverse correlation with an increase in oxytocin level (*r* = −0.68, *p* = 0.0005); subjects with ASD showed no similar correlation with a decrease in beta rhythm (observed for all types of tactile stimuli) (0.33 > *r* > 0.08).

Correlations between an oxytocin increase in and changes in PAF during the stimulation were noticed in both groups: for the control group, an increase in oxytocin was accompanied by a PAF decrease in all regions for a C-optimal stimulus (*r* < −0.65, *p* < 0.0009), and for subjects with ASD, the increase in oxytocin was accompanied by an increase in PAF in all regions for all types of tactile stimuli (*r* > 0.62, *p* < 0.004).

## 3. Discussion

The findings of the research indicate that individuals with ASD had lower salivary oxytocin capacity compared to the control group; however, their oxytocin level increased significantly during the experiment. The typical participants maintained a high level of oxytocin with no significant changes related to the procedure. Lower oxytocin level capacity in individuals with ASD was previously reported by numerous studies [54]. Some researchers hypothesized that reduced functioning of the oxytocin system that provides social reward learning could be responsible for the challenges in social communication and interaction in subjects with autism [55,56,57]. Tactile stimulation and, specifically, C-tactile afferent stimulation have been shown to be two of the triggers for the oxytocin release [58]. An increase in oxytocin level during affective tactile interactions correlated with reduced physiological arousal and the positive emotions associated with stimulation [20,59].

The key result of the current study is the atypical association between enhanced oxytocin level and the subjective assessment of the perceived tactile stimuli in participants with ASD. In particular, oxytocin release in subjects with ASD, unlikely in TD participants, did not correlate with the pleasantness of tactile stimuli, but was associated with a higher arousal score during the perception of stimuli. Moreover, our results demonstrate that the controls assessed all tactile stimuli, and above all C-tactile afferent stimulation, as significantly more pleasant than the participants with ASD. According to the previous findings, we suggest that an increase in oxytocin level after the stimulation in the ASD group could be attributed, rather, to the stress-induced response than to positive emotions caused by pleasant sensations [60,61,62]. The EEG findings also support the theory of stress-induced oxytocin release in subjects with ASD. In previous studies, we applied manual tactile stimulation with brushes of different softness and ticklishness to TD adults. We showed that the decrease in PAF was associated with a higher subjective pleasantness of touch and increased saliva oxytocin after the tactile stimulation [20]. According to the recent findings, the tactile stimulation delivered by the robotic device induced a significant increase in the PAF in both groups of subjects compared to the resting state. However, the correlation between the increase in the salivary oxytocin and the decrease in the PAF in the typical participants remained the same: the several participants of the control group who had an increase in the oxytocin level of more than 30% demonstrated a lower PAF during tactile stimulation compared to the resting state. At the same time, the vast majority of the typical participants with the higher basic oxytocin capacity compared to the ASD group did not show oxytocin release during the study. This result was in good agreement with the subjects’ reports. They claimed that they had not experienced a feeling of relaxation or pleasure usually accompanied by the decrease in PAF during massage procedures, meditation, or pleasant tactile stimulation [63,64,65]. Moreover, according to the self-reports of several individuals who participated in both our studies, tactile stimulation delivered by a human was more pleasant than stimulation presented by a robot, even though the stimuli were similar in their physical characteristics. These findings were consistent with the knowledge obtained from the investigation of the C-tactile system as the system which supports the appearance of positive emotions during social tactile contact between two individuals [66,67].

Another significant result of the study is the finding of an increase in antioxidant capacity in some participants during the study, and no similar changes in the other half of the sample. The increase in antioxidant capacity was not related to the pleasantness of the tactile stimulation but depended on the cognitive tension of the subjects during the experiment. The EEG changes corresponding to the increase in antioxidant capacity during the experiment included a decrease in theta rhythm mainly in the central regions, an increase in FF at rest, and a lower amplitude of the ERP components when presented with tactile stimuli. The observed EEG changes indicate a decrease in cognitive activity and mental tension during the experiment, which resulted, according to our hypothesis, in an increase in the antioxidant capacity of saliva. Thus, an increase in theta rhythm was considered to be functionally relevant for cognitive processing [68,69]. Recently, more and more researchers have linked the intensity of theta rhythm in central and parietal areas to the function of cognitive control [70]. According to different researchers, an increase in FD in a rest state is associated with the cognitive performance of tasks solved before [71] the subjective success of volunteers. Other researchers have shown that a decrease in FD at the very end of the study is associated with task-solving success and is indicative of satisfaction and less need for exertion [72,73]. The lower amplitudes of the P300 component observed in subjects with increased antioxidant capacity (Yes group) also indicate a lower level of cognitive strain during the study. For example, many researchers attribute lower P300 component amplitudes to decreased attention, memory, cognitive control, and, in general, to reduced cognitive strain and stress [74,75,76].

## 4. Materials and Methods

### 4.1. Participants

We recruited 21 subjects with ASD (13 females and 8 males) and 23 neurotypical subjects (14 females and 9 males). The diagnosis was confirmed with AQ screening and ADOS-2 methods by a certificated clinical psychologist in Our Sunny World center for ASD therapy. The inclusion criteria were the following: age from 20 to 40 y.o.; right-handed; no history of neurological or mental disorders (other than ASD for the target group); no history of a traumatic brain injury; participants did not take any antipsychotics, nootropics, antidepressants, or other medication; did not drink alcohol for less than 72 h before the study; and did not smoke for at least 4 h before the study. All participants with ASD had education levels similar to the control group. The exclusion criteria were ADOS-2 score ≥ 2 for the control group and ≤ 7 for participants with ASD. See Table 4 for details.

**Figure 6:**
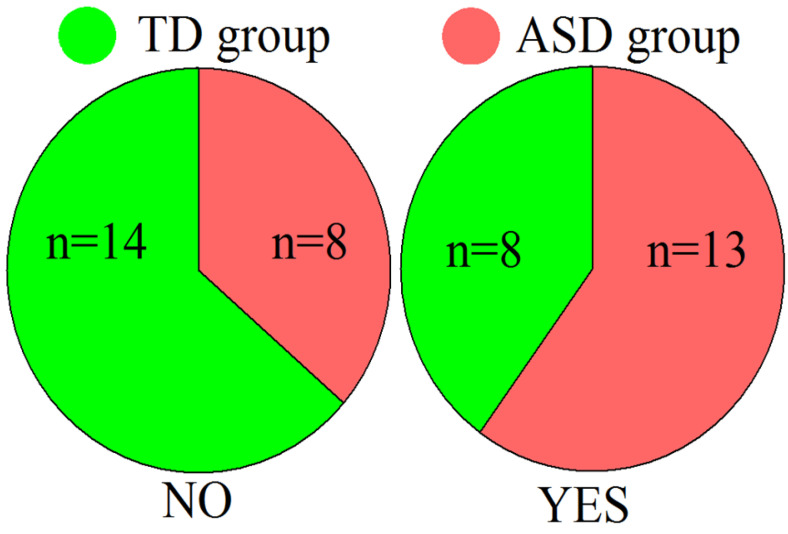
Diagram of distribution between ASD and TD group inside Yes and No groups.

When evaluating the level of antioxidants in saliva, we found that an increase of more than 30% was found in both participants with ASD and in the control group. All participants were divided into two groups: those in which there was a significant increase in antioxidants (greater than 30% of baseline—34 to 115%)—Yes, and those in whom the increase in antioxidant levels did not exceed 20% (changes from −17 to 11%). One participant with a 27% increase in antioxidant levels was excluded due to the uncertainty of their response. The distribution of the number of ASD and TD participants in the Yes/No groups is shown in Figure 6.

### 4.2. Stimulation and Data Acquisition

**Figure 7:**
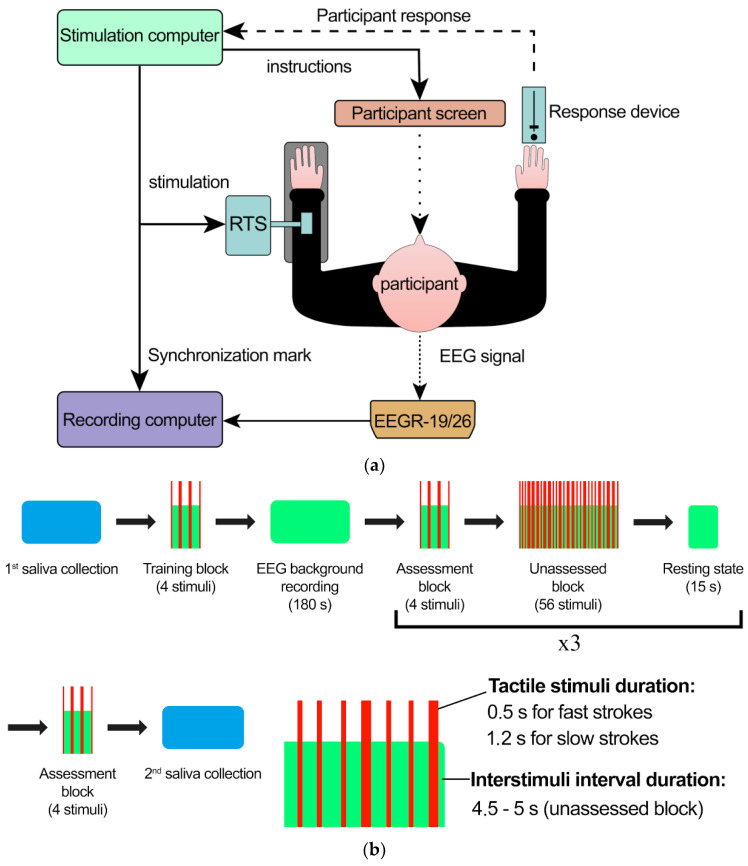
(**a**) Data acquisition setup; (**b**) experiment design. EEGR-19/26—EEG amplifier; RTS—rotary tactile stimulation system.

We used four types of tactile stimulation: strokes with a brush made of synthetic mongoose fur with two different velocities (5 cm/s and 30 cm/s) and two different forces (0.8 N and 0.075 N) in a pseudorandom order. The brush strokes were delivered with a custom-built robotic device (rotary tactile stimulator, RTS; Dancer Design, Ingleton, UK) driven by LabVIEW 8.6 (National Instruments, Austin, TX, USA) software. The RTS is able to present tactile stimuli with a specified velocity and force. The stimuli were presented in an experimental chamber, where participants were seated on a chair in front of a 21” display and to the right of the RTS, with their left arm immobilized comfortably on a vacuum cushion, which conforms to the shape of a hand and provides a comfortable position while minimizing forearm movements. The setup is visualized in Figure 7a.

After EEG cap application, the first saliva sample was collected and the training block followed: we presented 4 brush strokes using the RTS (0.8 N force and 5 cm/s velocity, 0.8 N force and 30 cm/s velocity, 0.1 N force and 5 cm/s velocity, and 0.1 N force and 30 cm/s velocity) and asked the participants to assess each stimulus the first time in terms of their pleasantness, ticklishness, and arousal. During this part of the study, the participants had the opportunity to ask any questions and received instructions on the following experimental part and proper use of the response device. The responses given during this block were not recorded and the training block was excluded from further analysis.

After the training block, the participants put in earplugs, which suppressed external sound signals but allowed them to hear the operator’s commands. The subjects took the earplugs out after the experiment was over. The recording of the background EEG signal was performed before the stimulation and lasted for 3 min (1.5 min with closed eyes and 1.5 min with open eyes). First, the operator gave the participant a voice command asking them to close or open their eyes, immediately after which, using a sensor, the operator manually sent a synchronization marker from the console. The experimental part consisted of 4 short rating blocks and 3 long blocks placed between them. The experiment always began with a short rating block. The short blocks were similar to the training blocks: they consisted of 4 brush strokes (0.8 N force and 5 cm/s velocity, 0.8 N force and 30 cm/s velocity, 0.1 N force and 5 cm/s velocity, and 0.1 N force and 30 cm/s velocity). Each stroke was rated by the subjects on three scales: pleasantness, ticklishness, and arousal. Between these short rating blocks, there were three long blocks of stimuli (56 stimuli in each block and 14 stimuli of each type). During the long block, the participants were instructed to sit in silence with their eyes closed. The interstimulus interval in the long blocks was randomized to be 4.5–5 s. After the end of the long block and the beginning of the short block, there was an interval of 15 s, during which no stimuli were given. During this time, the operator used his voice to ask the participant to open their eyes. A total of 184 brush strokes were acquired. After the stimulation, rest-state EEG was recorded, and a second sample of saliva was collected. The experiment scheme is shown in Figure 7b.

### 4.3. Stimuli Assessments

Participants rated touches on three analog scales:

- Pleasantness scale (unpleasant—pleasant).
- Tickling scale (not tickling—very tickling).
- Arousal scale (calming—excitement).

Answers to each question were given with the response device and with a visual analogue scale (VAS), which was displayed on the screen of the respondent. The response device was a combination of a range slider and an answer button; slider positions were recorded in the range from 0 (all the way down) to 10 (all the way up) with a step of 0.1. The participants did not see any numerical values on the scales. The position of the response device slider was displayed on the VAS; the participant confirmed their answer by pressing the button on the response device. Participants were not limited in time in evaluating their sensory and emotional impressions since the next stimulus was presented 1500 ms after the answer to the last question.

The participants rated stimuli during the training block before the experiment and during the short blocks. Before going through the training block, the participants were shown a graphic instruction on the screen, explaining the essence of the task and the mechanism of work of the response device. During short blocks, the first scale appeared on the screen of the respondent after the touch. The scales were always shown in the same order (emotion rating, tickling scale, and emotional activation scale); the next scale appeared after the participant rated the touch on the previous scale. The next stimulus was presented after the participant rated the previous stimulus.

### 4.4. Saliva Collection

Since saliva parameters depend on the time of day, samples were taken on an empty stomach from 10 to 12 a.m. Hygienic procedures of the oral cavity and the drinking water intake were carried out at least two hours before the study. The participants rinsed their mouth twice with cool drinking water before collecting saliva. Saliva was collected by spitting for 15 min. As a result, at least 1.5 mL of saliva was collected. The samples were centrifuged for 15 min at 2000× *g* at room temperature. Antioxidant activity was determined immediately after centrifugation. To determine oxytocin, the samples were stored at −20 °C for no more than a month.

### 4.5. Salivary Oxytocin Assay

Salivary oxytocin was quantified using the Oxytocin ELISA (Enzo, New York, NY, USA) as described in the instructions. The samples were stored at −20 °C for no more than a month after sampling. The samples were centrifuged at 13,400× *g* rpm for 5 min using an Eppendorf MiniSpin (Eppendorf AG, Hamburg, Germany). If necessary, the samples were diluted 1:1, 1:3, and 1:9 with an assay buffer kit to a volume of 100 µL. All the samples were analyzed in duplicates. Samples and standard solutions were added to 96-well plates along with polyclonal antibodies to oxytocin and oxytocin conjugate (alkaline phosphatase) and incubated for 24 h at 4 °C. The plate was then washed and incubated with the substrate for 1 h at room temperature, and the absorbance was measured with a MultiScan GO plate reader (Thermo Fisher, Waltham, MA, USA) at 405 nm with a correction between 570 and 590 nm. The analytical signal was calculated using the formula [(A405–A570) + (A405–A590)]/2. The calculated values were analyzed using the ElisaAnalysis software (ELISA Analysis, elisaanalysis.com accessed 26 November 2020).

### 4.6. Salivary Antioxidant Capacity Assay

The antioxidant capacity of saliva was quantified using a chemiluminescent system based on a free radical source 2,2′-azobis(2-amidinopropane) dihydrochloride (ABAP, Sigma, St. Louis, MO, USA) and luminol (Sigma, St. Louis, MO, USA) as a chemiluminescent probe. Briefly, a solution of luminol 1 mmol/L and ABAP 50 mmol/L in phosphate-buffered solution (100 mM KH_2_PO_4_, pH 7.4, Sigma, St. Louis, MO, USA) was placed in a 37 °C chemiluminometer cuvette. Chemiluminescence was recorded to a steady-state level. Next, 200 µL of saliva was added to the cuvette. Chemiluminescence was recorded to a new steady-state level. The antioxidant capacity was calculated as the depression area of the chemiluminescence. The mean *S* value and standard deviation were calculated from triplicates. The measurements were carried out on a 12-channel Lum-1200 chemiluminometer (DISoft, Moscow, Russia). Signal processing was performed using the PowerGraph 3.3 Professional program (DISoft, Moscow, Russia).

### 4.7. Salivary Indexes

For assessing changes in parameters before and after experiments, we calculated relative indices for oxytocin and antioxidant capacity as follows:

(1) Oxytocin index, OI = (oxytocin after experiment—oxytocin before experiment)/oxytocin after experiment;
(2) Antioxidant capacity index, AI = (antioxidant capacity after experiment—antioxidant capacity after experiment before experiment)/antioxidant capacity after experiment after experiment.

### 4.8. EEG Recording and Preprocessing

Electroencephalogram data were acquired using a 19-channel EEG recording system “Encephalan” (Medicom MTD, Taganrog, Russian Federation) and 19-electrode caps with electrodes placed according to a modified international 10–20 system (Fp1, Fp2, F7, F3, Fz, F4, F8, T3, C3, Cz, C4, T4, T5, P3, Pz, P4, T6, O1, and O2). Averaged mastoids (A1 and A2) were used as the reference. The bandpass filter during the recording was set to 0.1–30 Hz, and the sampling rate was 250 Hz.

EEG data were processed with MATLAB R2017b (MathWorks Inc., MA, USA) with the EEGLAB toolbox (https://eeglab.org/). Ocular movement artifacts were removed with Independent Component Analysis (ICA), and remaining movement-related and EMG-related artifacts were removed according to amplitude criteria and visual inspection.

### 4.9. EEG Analysis

The following EEG parameters were calculated:

The power spectral density. The data were transformed using FFT, and power spectral density (PSD) was calculated for the frequency bands of interest: delta (2–4 Hz), theta-1 (4–6 Hz), theta-2 (6–8 Hz), alpha-1 (8–10 Hz), alpha-2 (10–13 Hz), and beta (13–20 Hz). The PSD values were log-transformed for further analyses.

The peak alpha frequency. To calculate the PAF, we selected EEG epochs from the resting states (initial closed-eyes resting state and interstimulus interval for each three experimental blocks) and epochs beginning from a tactile stimulus onset to its offset. The calculations were made with MATLAB (MathWorks) using the hamming window with 50% overlap between contiguous sections for each trial separately, and then averaged. PAF identification was conducted using FFT. The PAF was estimated for each experimental condition as a value of frequency with the maximal PSD from an 8–13 Hz range based on the frequency discretization data. If no peak was present, it was not counted.

Fractal dimension. We calculated the fractal dimension (FD) using the Higuchi algorithm. The FD was computed for each EEG channel and for each analyzed EEG fragment.

### 4.10. ERP Analysis

For ERP analysis, the EEG recordings were filtered in the 0.1–30 range with additional 50 Hz notch filtering. Muscular and eye movement artifacts were removed with ICA, and occasional artifacts were removed manually. After that, the recordings were epoched in the range of −1000–5500 ms relative to the moment of stimulation onset. Each epoch was checked for artifacts manually by two independent experts. Each subject had more than 70% artifact-free epochs. Then, the epochs were averaged, and the grand averages for all the conditions were obtained. All the operations were made with Brain Vision Analyzer 2.2.0.7383 (Brain Products GmbH, Gilching, Germany). The latencies and amplitudes of ERPs were extracted and analyzed with Statistica 10 (Stat Soft Inc., Tulsa, OK, USA). Data were standardized, checked for normality with a Kolmogorov–Smirnov test, and further analyzed with an analysis of variance (ANOVA) and pairwise Student *t*-tests for single electrodes.

### 4.11. Statistical Analysis

Statistical analysis was conducted with STATISTICA version 13. Differences between groups were calculated using the repeated measures ANOVA with Bonferroni post hoc comparison (*p* < 0.05). The effect was computed for the ERP amplitudes and latencies and FD, PAF, and PSD. To reduce the number of comparisons, we applied statistical analysis for the following sites: F3, Fz, F4 (frontal topography), C3, Cz, C4 (central), and P3, Pz, P4 (parietal). Degrees of freedom for F-ratios were corrected with the Bonferroni method. Spearman’s rank correlation was used to evaluate the relationships between EEG values, stimuli assessments, and saliva capacity (*p* < 0.05). All analytical steps were performed with STATISTICA version 13 (StatSoft), and scripts were implemented in MATLAB R2018b (MathWorks, Natick, MA, USA).

## 5. Conclusions

According to our findings, an increase in oxytocin level was observed only in ASD participants and was associated with a stress-induced response evoked by tactile stimulation, which was accompanied by a PAF increase. An increase in antioxidant capacity was observed in some ASD participants, as well as in some subjects in the control group, it was accompanied by a lower amplitude of the P300 component when presented with tactile stimuli and a decrease in the theta rhythm power and an increase in the resting-state FD. The latter EEG changes, according to the literature, accompanied states of satisfaction after successful task performance and decreased cognitive control.

### Limitations

First, the groups were relatively small. It is especially important as autism is a spectrum with as-yet-understudied internal variation.

Stress levels in subjects were not assessed, nor were vegetative responses (pulse rate/variability, skin conductance, and/or respiratory rate). Including these traits could be advised for further research.

## Figures and Tables

**Table 1:** Descriptive statistics of stimuli assessments in subjects of TD and ASD group and differences between them.

		Asperger’s				Control Group			
	Stimuli (sm/s-N)	Mean	Std. Dv.	*t*	*p*	Mean	Std. Dv.	*t*	*p*
**Pleasantness (1–9)**	5–0.1	4.14	1.61	−2.63	0.017	3.84	1.14	−8.74	0.000
	5–0.8	5.51	1.13			7.17	1.39		
	5–0.1	4.14	1.61	−0.99	0.335	3.84	1.14	−3.00	0.007
	30–0.1	4.63	1.04			5.24	1.39		
	5–0.1	4.14	1.61	−1.24	0.231	3.84	1.14	−3.87	0.001
	30–0.8	4.79	1.34			5.51	1.24		
	5–0.8	5.51	1.13	2.61	0.018	7.17	1.39	4.97	0.000
	30–0.1	4.63	1.04			5.24	1.39		
	5–0.8	5.51	1.13	2.88	0.010	7.17	1.39	4.49	0.000
	30–0.8	4.79	1.34			5.51	1.24		
	30–0.8	4.79	1.34	0.57	0.576	5.51	1.24	1.59	0.126
	30–0.1	4.63	1.04			5.24	1.39		
**Tickle (1–9)**	5–0.1	3.84	1.14	4.32	0.000	3.64	2.55	3.28	0.004
	5–0.8	7.17	1.39			2.02	1.94		
	5–0.1	3.84	1.14	1.75	0.098	3.64	2.55	−0.61	0.551
	30–0.1	5.24	1.39			3.84	2.46		
	5–0.1	3.84	1.14	2.63	0.018	3.64	2.55	3.41	0.003
	30–0.8	5.51	1.24			2.21	1.68		
	5–0.8	1.94	1.77	−3.35	0.004	2.02	1.94	−3.75	0.001
	30–0.1	3.19	2.49			3.84	2.46		
	5–0.8	1.94	1.77	−2.07	0.054	2.02	1.94	−0.70	0.493
	30–0.8	2.60	2.00			2.21	1.68		
	30-0.1	3.19	2.49	1.64	0.120	3.84	2.46	4.51	0.000
	30–0.8	2.60	2.00			2.21	1.68		
**Arousal (1–9)**	5–0.1	4.17	2.16	2.59	0.019	3.71	2.22	2.74	0.012
	5–0.8	3.17	2.13			2.60	1.67		
	5–0.1	4.17	2.16	−0.54	0.597	3.71	2.22	−3.10	0.005
	30–0.1	4.27	2.06			4.31	2.16		
	5–0.1	4.17	2.16	0.00	1.000	3.71	2.22	−0.78	0.443
	30–0.8	4.17	1.74			3.94	1.96		
	5–0.8	3.17	2.13	−2.87	0.011	2.60	1.67	−4.76	0.000
	30–0.8	4.27	2.06			4.31	2.16		
	5–0.8	3.17	2.13	−3.35	0.004	2.60	1.67	−3.55	0.002
	30–0.8	4.17	1.74			3.94	1.96		
	30–0.1	4.27	2.06	0.51	0.620	4.31	2.16	1.96	0.063
	30–0.8	4.17	1.74			3.94	1.96		

**Table 2:** The descriptive statistics of the salivary oxytocin and antioxidant capacity in TD group and ASD group. AI—antioxidant index. OI—oxytocin index.

Saliva Parameter		N	Mean	Lower Quartile	Upper Quartile	Std. Dev.
ASD Group						
**Antioxidant**	**First probe**	21	988.3	626.3	1312.7	477.2
	**Second probe**	21	1479.0	796.3	1786.8	787.9
	**AI**	21	0.6	0.2	0.9	0.6
**Oxytocin**	**First probe**	21	229.4	96.8	354.9	162.9
	**Second probe**	21	288.2	149.9	396.4	170.6
	**OI**	21	0.467	−0.059	0.615	0.809
**TD Group**						
**Antioxidant**	**First probe**	23	1047.8	707.51	1251.70	445.57
	**Second probe**	23	1169.3	727.80	1494.29	490.77
	**AI**	23	0.2	−0.04	0.22	0.42
**Oxytocin**	**First probe**	23	430.2	331.11	534.67	138.47
	**Second probe**	23	422.5	323.47	548.39	143.22
	**OI**	23	−0.017	−0.034	0.030	0.097

**Table 3:** Post hoc analysis Bonferroni test—*p*-values: repeated measures ANOVA for group with enhanced antioxidant capacity (YES) and without it (NO)—time(4) × group effect for theta rhythm power spectrum density (PSD) and fractal dimension (FD).

**Intergroup** **Differences**	**EEG Features**	**Resting State**	**Block 1**	**Block 2**	**Block 3**
	**PSD 4–8 Hz**	1	0.0717	0.0006	0.0000
	**FD**	0.0972	0.9539	1	0.4578
**intragroup differences between time blocks: PSD 4–8 Hz**					
**Group**	**“rest-b1”**	**“rest-b2”**	**“rest-b3”**	**“b1−b2”**	**“b2−b3”**
**Yes**	1	0.0009	0.0000	0.0027	0.0000
**No**	0.1474	0.5568	0.0717	1	1
**intragroup differences between time blocks: FD**					
**Group**	**“rest-b1”**	**“rest-b2”**	**“rest-b3”**	**“b1−b2”**	**“b2−b3”**
**Yes**	0.8978	0.0005	0.0000	0.1304	0.0000
**No**	1	1	1	1	1

**Table 4:** Demographic data of study participants by group.

Group	N	Gender			Age	AQ	TEAQ
TD		F	14	Mean	27.7	17.8	133.3
	23						
		M	9	SD	7.9	4.3	21.9
ASD		F	13	Mean	28.6	38.6	96.6
	21						
		M	8	SD	9.22	5.0	16.5
Yes		F	14	Mean	27.3	31.7	112.7
	21 *						
		M	7	SD	7.3	5.8	26.4
No		F	14	Mean	28.4	27.5	116.8
	22 *						
		M	8	SD	8.5	5.8	22.9

* One subject was excluded due to the uncertain antioxidant response.

## Data Availability

The data presented in this study are available on request from the corresponding author.
